# Preparing for the future of medical education over the next five years: the key recommendations of an expert panel

**DOI:** 10.5116/ijme.6848.3936

**Published:** 2025-06-25

**Authors:** Shabih Zaid, Shahid Hassan, John Sandars, Hasnain Baloch, Tabassum Zehra, Azim Mirzazadeh, Madalena Patricio

**Affiliations:** 1 Al -Sadiq International Virtual University (SIVU), USA; 2 American University of Barbados School of Medicine, Barbados; 3 School of Medicine, Edge Hill University, UK; 4 IMU University, Malaysia; 5 Medical Education and Informatics Department, Sultan Qaboos University, Oman; 6 Tehran University of Medical Sciences (TUMS), Iran; 7 University of Lisbon, School of Medicine. Portugal

**Keywords:** Medical education, future planning, strategic planning, Institutional change

## Introduction

Heraclitus, the Ancient Greek philosopher, stated that “everything flows, nothing stands still”.^1^   This quotation highlights the constant evolution, and some would say revolution, in technological, social and scientific developments. In response to these evolving trends, medical education will also need to evolve if it is to be ‘fit for purpose’.  This evolution will require academic and institutional renewal, and strategic planning is an essential process for this effective evolution and renewal.^2^  Strategic planning is a two stage organisational development process in which first, a clear vision is developed to predict future external conditions and this vision is used to create several recommendations for changes that will be required to effectively respond to future conditions;  and second, the implementation of the proposed changes, including evaluation and modification to ensure that the changes are being effective to the evolving external conditions.^2^

In this article, we present several key recommendations that can serve as a practical guide for medical educators in shaping their strategic plans for providing medical education over the next five years.

### Development of the key recommendations

The key recommendations for providing medical education over the next five years were developed by an expert panel of scholars in medical education and are based on their presentations at an online symposium organised by al-Sadiq International Virtual University (SIVU).

The expert panel method is widely used to inform key recommendations for future policy and strategic planning, with the intention to identify key common themes across the opinions of several experts.^3^  This method has a breadth of approaches , from consensus and evidence-based to the collation of expert opinion.^3^ Expert opinion is developed through personal and collective experience developed over many years within a specific area of interest through the integration of their extensive range of experiences as a scholar, such as discussions with other scholars, attendances at conferences and a wide general  awareness of current research and practice.^4^  

The panel members were identified as internationally renowned scholars in medical education by the lead conference organiser (SZ) and the symposium’s organising committee. Each scholar was selected based on their significant contributions to medical education, including authorship of influential peer-reviewed publications, conference presentations, and leadership roles in academic institutions, professional bodies and accreditation agencies. The panel members were: (a) Professor Shahid Hassan:  Associate Dean Academic at American University of Barbados School of Medicine, Barbados; (b) Professor John Sandars: Professor of Medical Education, School of Medicine, Edge Hill University, United Kingdom; (c) Mr Hasnain Zafar Baloch:  Senior Manager, e-learning, International Medical University (IMU), Malaysia; (d) Dr Tabassum Zehra:  Assistant Professor, Medical Education and Informatics Department, Sultan Qaboos University, Oman; (e) Professor Azim Mirzazadeh: Professor of Departments of Internal Medicine and Medical Education, Tehran University of Medical Sciences (TUMS), Iran and President of Iranian Society of Medical Educationists (IRSOME); and (f)  Professor Madalena Patricio, Emeritus Professor of Medical Education, University of Lisbon School of Medicine, Portugal, former President of Association for Health Professions Education (AMEE) and Member of the United Nations Educational, Scientific and Cultural Organization (UNESCO) International Council of the Education Department in Bioethics.

Each member of the panel was independently invited to provide a 15-minute presentation on a specific aspect of medical education with the content based on their expert opinion of the key recommendations for providing medical education over the next five years.

All presentations were delivered online and recorded by Zoom. The recordings were also transcribed, and the key recommendations were identified by the Meeting Summary with Zoom AI Companion function. The identified key recommendations were returned to all presenters to check for any misalignment with their presentation and the corrected lists were collated by SZ and JS. The corrected final lists of key recommendations were returned to all presenters for any further corrections, and the final collated version was used to inform the writing of the article.  This approach to using artificial intelligence (AI) for qualitative information analysis followed best practice guidance, with advice that the technology can assist but does not replace human involvement.^5^

The members of the expert panel gave permission for their presentations to be recorded, and these presenters are also the co-authors of this article.  Formal ethical approval was not required since this is a scholarly collection of expert opinion and not research. The questions to the panel from the online global audience of the symposium were not recorded.

The key recommendations that we present can serve as a practical guide for medical educators in shaping their strategic plans for delivering medical education over the next five years.  This guidance can be useful for all stakeholders involved in providing medical education across its continuum from undergraduate to postgraduate training to continuing professional development. However, most of the practical recommendations are of particular relevance to those educators in medical schools with specific responsibility for curriculum design and delivery, such as programme and course managers, but also faculty developers and senior staff responsible for faculty development and institutional change. In addition, there are specific implications and recommendations for accreditation agencies.

### Curriculum design and delivery

Key recommendations for introducing interdisciplinary and team-based learning approaches, with increased personalised and flexible learning models and assessment approaches, was highlighted as being important to ensure that medical students have the appropriate knowledge and skills for practice in a future healthcare environment. Evolving developments in Artificial Intelligence (AI) was considered to be a major influence on learning and assessment approaches over the next five years, with recommendations to develop (a) a comprehensive digital competency framework for medical education that addresses both digital skills and health informatics, including ethical guidelines, and (b) maintain an essential focus on critical thinking and humanistic skills when AI is used for assessment.

There were also recommendations to integrate emerging topics, such as bioethics, climate change and precision medicine, in the medical curriculum, along with initiatives for supporting the wellbeing of all students.

### Faculty development and institutional change

Achieving the vision of curriculum renewal through design and delivery will require faculty development and institutional change.  A key recommendation for faculty development was upskilling faculty in using AI tools and digital technologies for teaching and assessment. For responsive institutional change, key recommendations included (a) developing a shared stakeholder  vision for the future trends in medical education with a long-term roadmap for change over five years that starts with small, closely monitored steps to motivate faculty members; (b) ensuring that there are comprehensive digital strategies and infrastructure to support integration of new technologies, including urgent upgrading of current technology infrastructures; and (c) evaluating the impact of new interventions and processes through research and quality improvement initiatives to inform further development and change.

### Implications for accreditation agencies

The provision of medical education is highly dependent on the standards required by accreditation agencies and key recommendations were (a) an urgent need to update accreditation standards to reflect emerging competencies, such as digital health, AI, and global health, and (b) to improve collaboration and harmonisation of standards at regional and international levels.

## Discussion

The recommendations that we have presented can usefully inform the provision of medical education over the next five years. Despite the different contexts of medical education and individual differences of the experiences and interests of the expert panel, there was an overall similarity. There was also an inter-relationship of the identified components of medical education that will need to be considered if medical education is to be ‘fit for the future’. These essential components span curriculum design and delivery, faculty development and institutional change, and accreditation agencies. All these components need to be considered and appropriately addressed if medical education is to evolve to meet the expected trends in technological, social and scientific developments.

The importance of AI for the future provision of medical education was highlighted, especially since it is becoming increasingly ubiquitous, with numerous applications for providing the curriculum. The applications range from learning content creation and delivery, to learning analytics for tracking performance and providing personalised education, to virtual reality simulations for developing essential clinical skills.^6^ Developing competencies in the effective and appropriate use of these technologies is an important recommendation and it has also previously been identified as a priority area for both medical educators and students.^7^

The importance of the holistic development of medical students was also highlighted, especially to ensure that there is a global workforce that can respond to major evolving global challenges, including universal health coverage and responding to the impact of climate, environmental and conflict changes. This area has also been previously emphasised,^8^ and continues to be of high importance.^9^

The implementation of recommendations is an essential aspect of strategic planning for changing medical education to be ‘fit for the future’. It is likely that progressive curriculum redesign will be required and whilst there is a desire for quick wins while waiting for larger curriculum changes, it is important to remember that different schools are at varying stages of progress and small adjustments may commence, especially through faculty development initiatives in which there is a shared vision for change.^10^ Major curriculum redesign in many contexts is likely to be highly influenced by recognising that the current curriculum needs change, such as through quality assurance evaluations,^11^ or by external pressures through accreditation.^12^  To reduce the daunting task of major curriculum redesign, increasing collaboration between medical schools may provide the impetus and support for implementing change.^13^

The strength of these recommendations lies in their development, which was informed by the presentations of six scholars in medical education from several global contexts, with each facing unique local challenges and systems related to health care, curricula, resources, faculty and students. Although no formal consensus approach was used, the process used was systematic and rigorous, with similar key recommendations identified across all the presentations and alignment with recent literature.

## Conclusions

Our world is in constant evolution, especially through technological, social and scientific developments. It is essential that medical education can also evolve to meet these challenges and beginning to engage with strategic planning can ensure that medical education is ‘fit for the future’. Strategic planning requires having a clear vision of future trends and then implementing changes to meet these trends. The intention of this article is to present several practical key recommendations made by an expert panel of scholars in medical education, with a focus on curriculum design and delivery, faculty development and institutional change and implications for accreditation agencies.

### Acknowledgments

We thank Sakina Naqvi for the organisation and technology support of the symposium and expert panel. We also thank Professor Qasim Jafry for introducing the Symposium, Professor Farzana Mahdi and Professor Sucheta Dandekar for acting as Co-chairs of the Symposium.

### Conflict of Interest

The authors declare that they have no conflict of interest.
